# Supplementary figures and images for: SERINC5 restricts influenza virus infectivity
Source: PLoS Pathog. 2022 Oct 12;18(10):e1010907. doi: 10.1371/journal.ppat.1010907 (PMC9591065; doi:10.1371/journal.ppat.1010907)

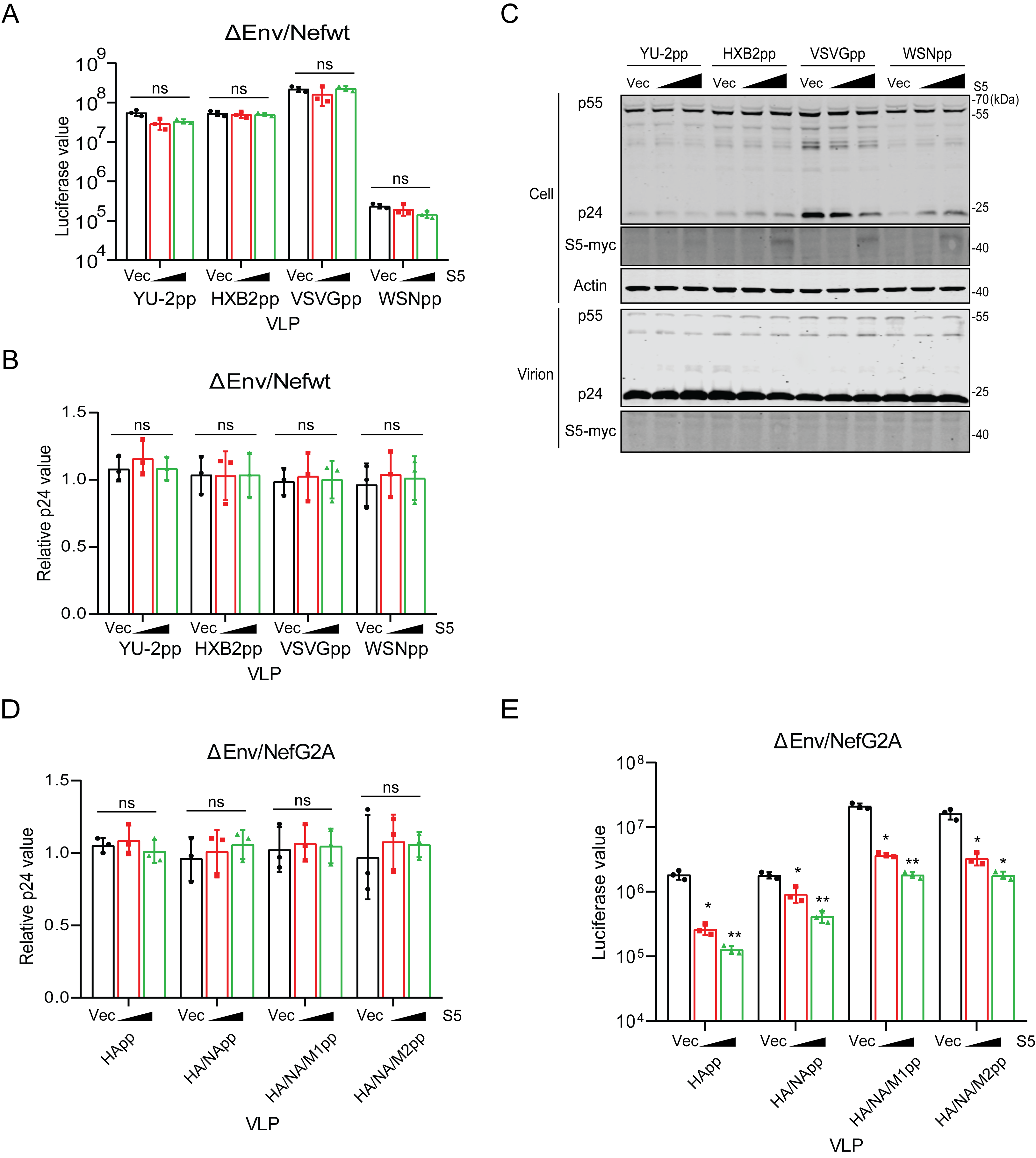

Supplement: S1 Fig — A-C: Pesudoviruses were produced using ΔEnv/NefWT HIV-1NL4-3 that contains the wild type Nef, bearing Env (HXB2), Env (YU-2), VSV-G, or HA/NA, in the presence of different amounts of SERINC5 plasmid DNA. Viral infectivity (A), viral particle production (B) and protein expression (C) were determined as described in Fig 1. D-E: Pseudoviruses were produced with the ΔEnv/NefG2A HIV-1NL4-3 plasmid together with IAV HA, NA, M1, M2 plasmids (pCAGGS-HA, pCAGGS-NA, pCAGGS-M1, pCAGGS-M2), in the presence of increasing amounts of SERINC5 DNA. Viral particle production (A) and viral infectivity (B) were measured as described in Fig 1. Results shown are the averages of three independent experiments. Statistical significance was calculated with the unpaired t-test. ns: not significant; *: P<0.05; **: P<0.01. (TIF) [file ppat.1010907.s001.tif]

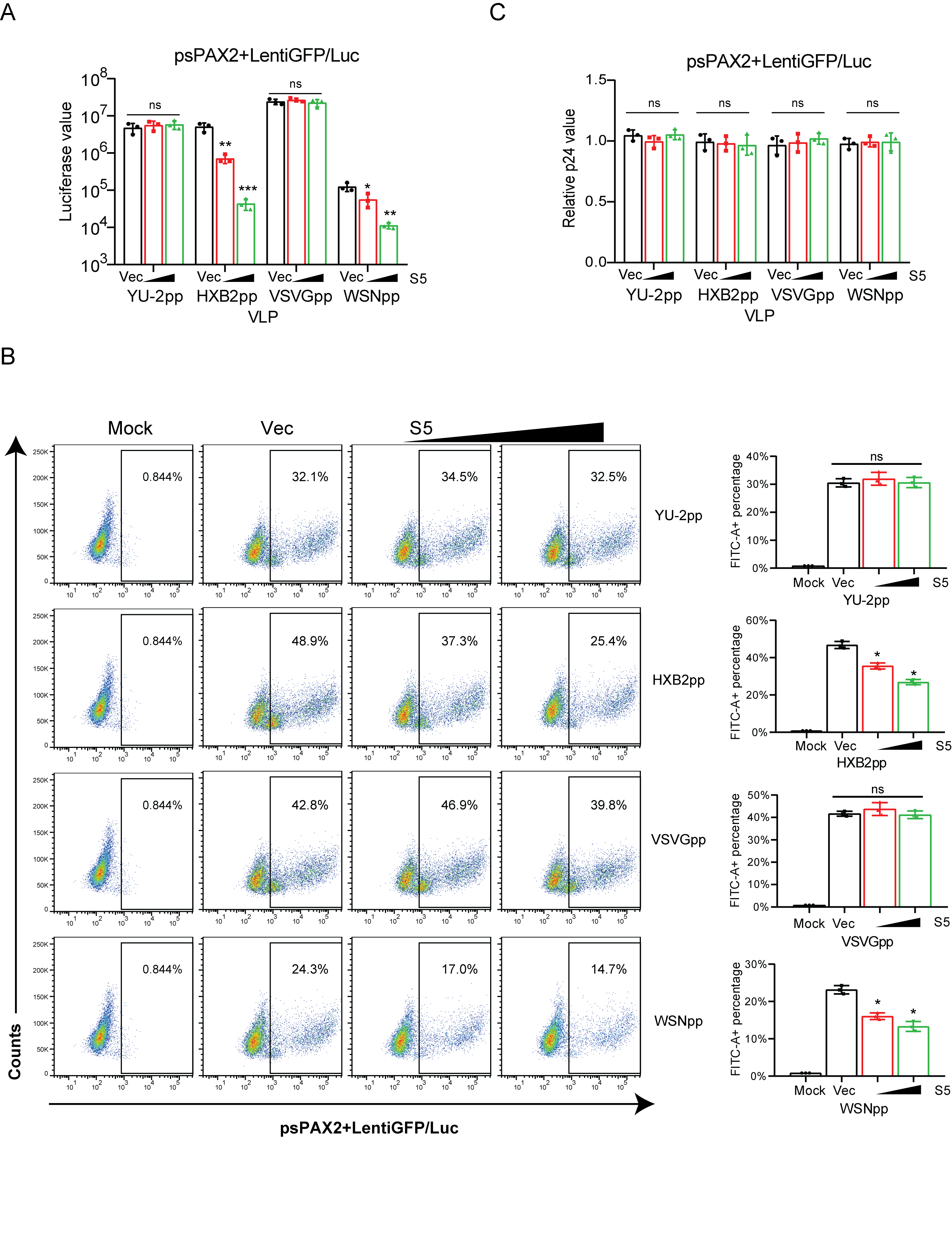

Supplement: S2 Fig — A-C: Pseudoviruses were produced with the psPAX2 plasmid that expresses viral Gag and Pol, LentiGFP/Luc that expresses luciferase reporter, pCAGGS-HA and pCAGGS-NA that express IAV HA and NA proteins, in the presence of increasing amounts of the SERINC5 plasmid. Equal amounts of viruses as normalized to the levels of p24Gag were used to infect the Hela-CD4+ cells in a 6-well plate at a density of 3×10^5^ per well. At 48 h after viral infection, half of the cells were lysed to measure luciferase activity (A). The other half cells were fixed to score FITC-positive cells by flow cytometry (B). (C) Viral production was determined by p24Gag ELISA. Results in this figure are the means from three independent experiments. Statistical significance was analyzed by unpaired t-test. ns: not significant; *: P<0.05; **: P<0.01; ***: P<0.001. (TIF) [file ppat.1010907.s002.tif]

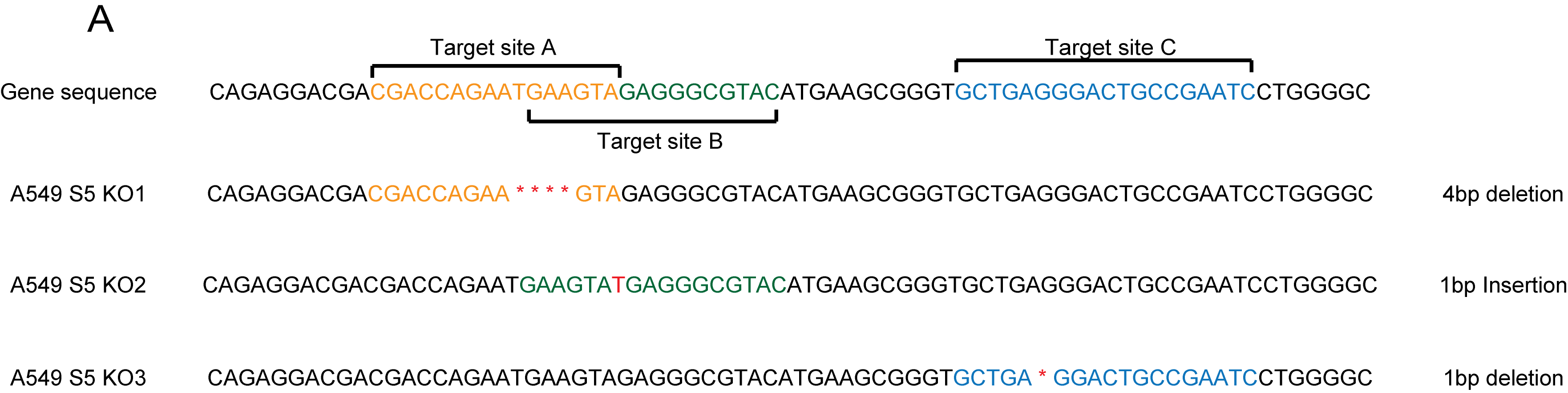

Supplement: S3 Fig — SERINC5 was knocked out with CRISPR/Cas9 in A549 cells. Single cell clones were selected with puromycin (0.8 ug/ml). Genomic DNA was extracted from the selected cell clones and amplified for exon 2 of SERINC5 gene. The PCR products were cloned and sequenced. (TIF) [file ppat.1010907.s003.tif]

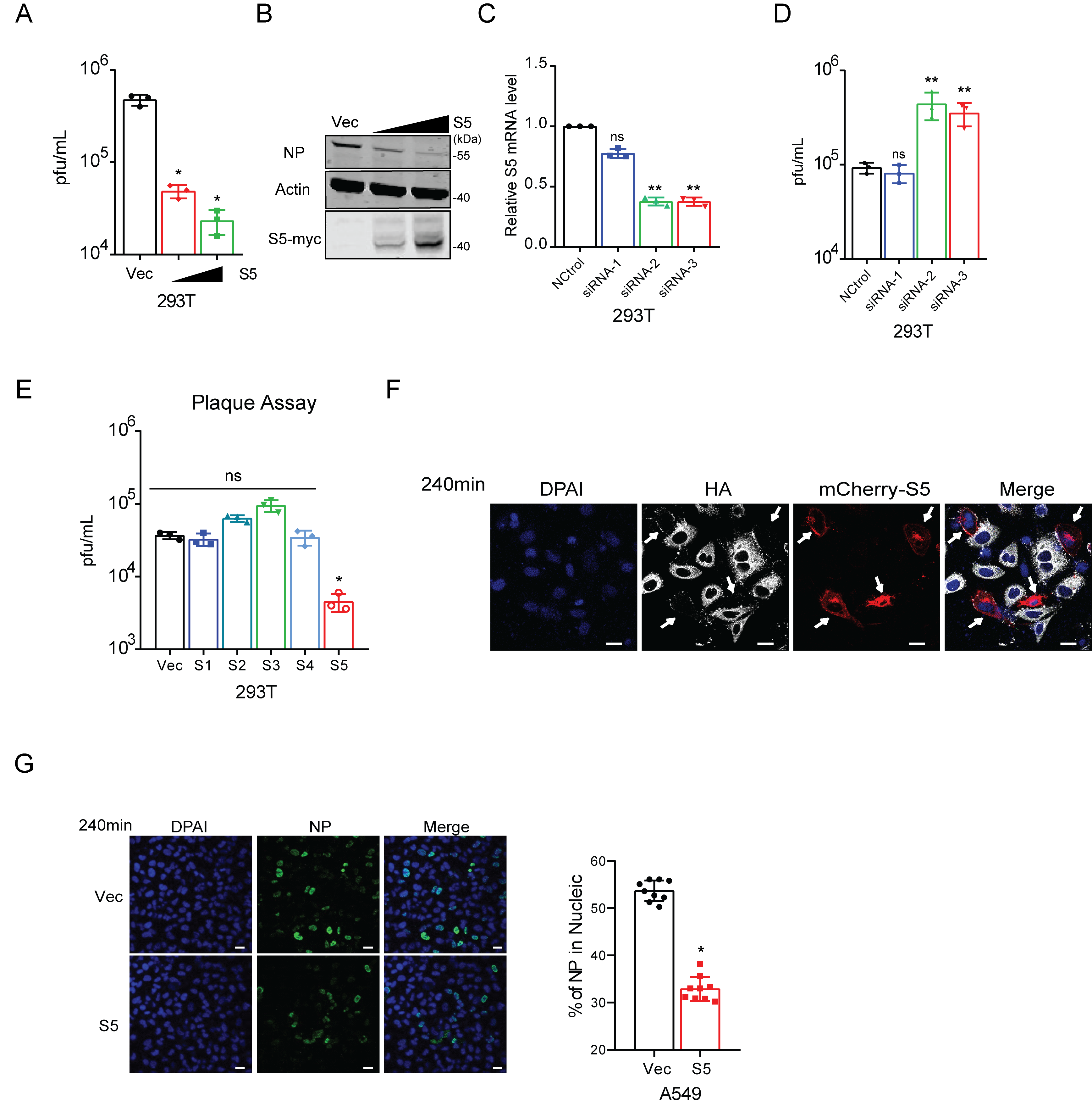

Supplement: S4 Fig — A-B: HEK293T cells were transiently transfected with SERINC5 DNA, then infected with A/WSN/33 (MOI = 0.05). Supernatants were harvested 24 h.p.i. Virus titers in the culture supernatants were determined by plaque assays. Levels of SERINC5, viral NP and actin in the infected cells were determined by Western blotting. C-D: HEK293T cells were transfected with SERINC5 siRNA, then infected with virus A/WSN/33 (MOI = 0.01). Viruses and infected cells were harvested 24 h.p.i. (C) SERINC5 mRNA level was quantified by RT-PCR. (D) Virus titers were determined by plaque assays. E: HEK293T cells were transfected with SERINC cDNA S1 to S5, then infected with WSN (MOI = 0.01) for 16 h. Titers of IAV in the supernatants were determined by viral plaque assays. F: Images of A549 cells that were transiently transfected with mCherry-SERINC5 and infected with WSN (MOI = 1) for 4 hours, stained for nuclei (blue), HA (white) and SERINC5 (red). Scale bar, 20 μm. G: Images of WSN (MOI = 1) infected, SERINC5-overexpressing cell lines at 4 h.p.i., stained for nuclei (blue) and NP (green). Scale bar, 20 μm. Statistical analysis is shown in the columns. Results shown are the averages of three independent experiments. Statistical significance was calculated by unpaired t-test. ns: not significant; *: P<0.05; **: P<0.01. (TIF) [file ppat.1010907.s004.tif]

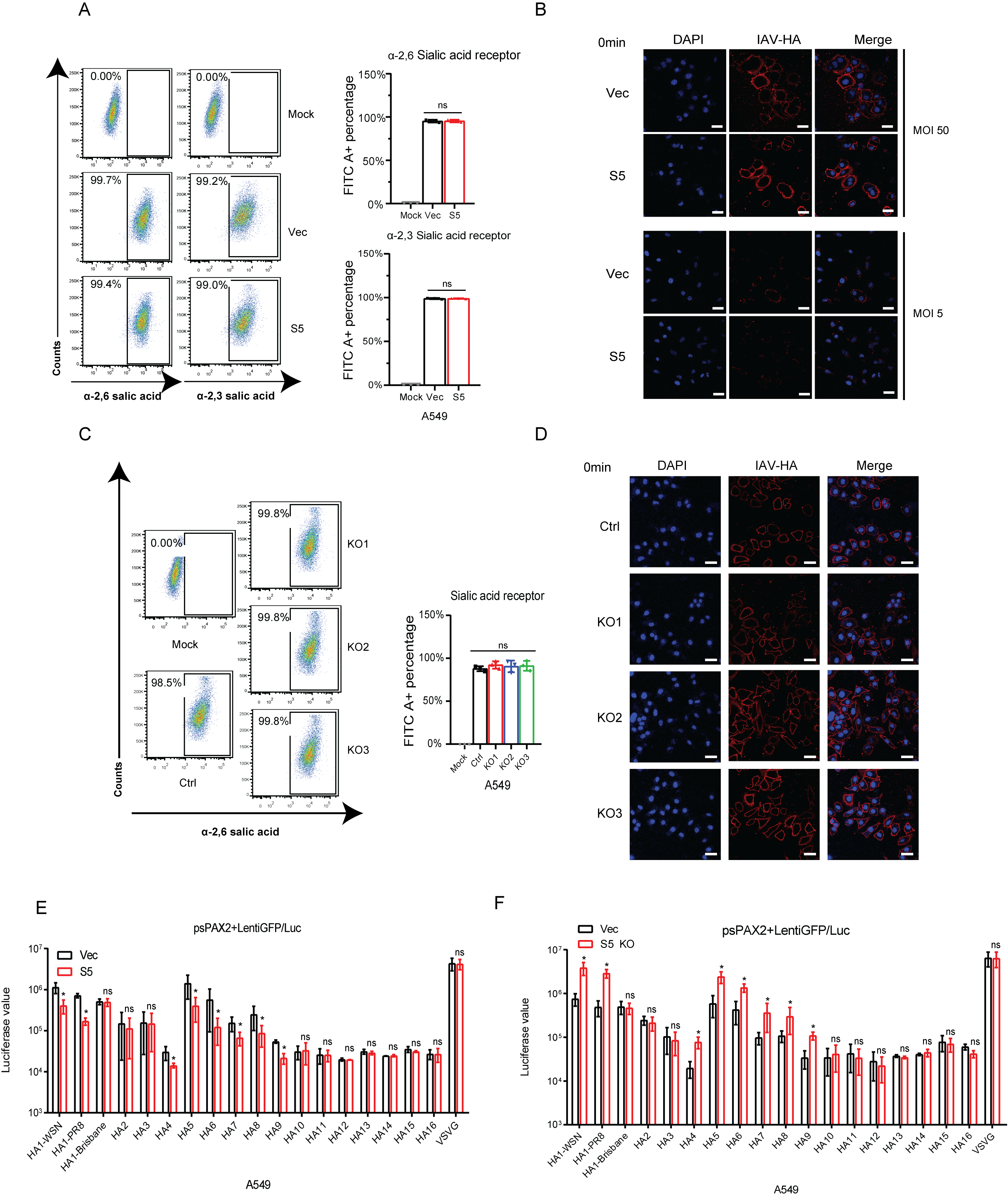

Supplement: S5 Fig — A: Levels of (α-2,6) and (α-2,3) sialic acid linkages at the surface of A549 SERINC5-overexpressing cells Cells were fixed and incubated with FITC-conjugated Sambucus nigra lectin (SNA) to detect (α-2,6) sialic acid linkages and biotinylated Maackia amurensis lectin II (MAL, Vector Labs #B-1265) to detect (α-2,3) sialic acid linkages, followed by streptavidin-FITC (Invitrogen SA1001). Percentages of FITC-positive cells are summarized in the bar graph. B: IAV binding to A549 SERINC5-overexpressing cells. After incubation with WSN (MOI = 50/5) at 4°C for 1 h, cells were fixed and stained for nuclei (blue) and HA (red). Scale bar, 30 μm. C: Levels of (α-2,6) sialic acid linkages at the surface of A549 SERINC5-knockout cells. Cells were treated in figure A. Percentages of FITC-positive cells are summarized in the bar graph. D: IAV binding to A549 SERINC5-knockout cells. After incubation with WSN (MOI = 50) at 4°C for 1 h, cells were fixed and stained for nuclei (blue) and HA (red). Scale bar, 30 μm. E-F: The original luciferase value in Fig 4A and 4B. Results shown represent three independent experiments. Statistical significance was calculated by unpaired t-test. ns: not significant; *: P<0.05. (TIF) [file ppat.1010907.s005.tif]

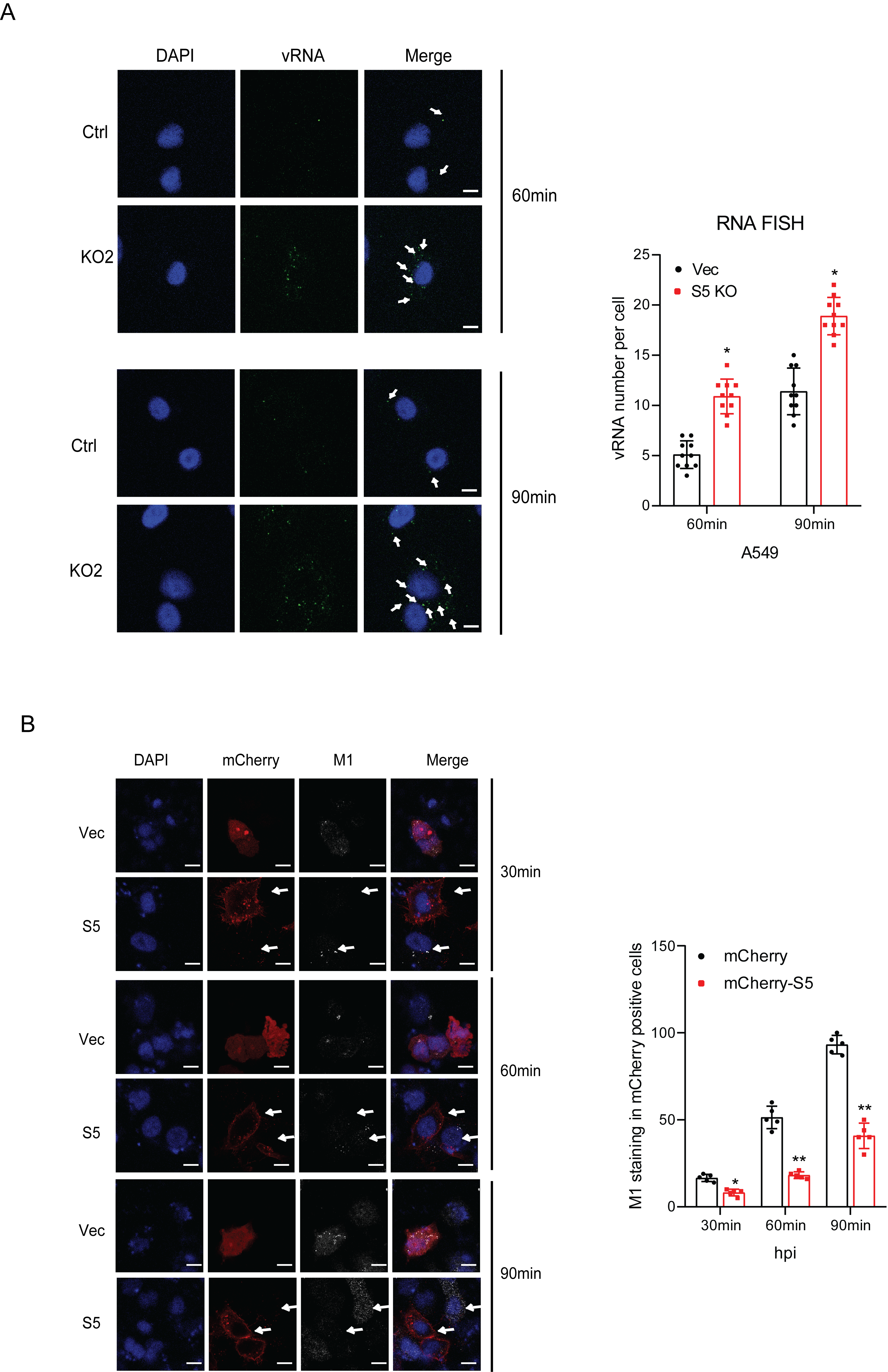

Supplement: S6 Fig — A: A549 SERINC5-knockout cells were incubated with IAV WSN at MOI = 20 for 60 min, or 90 min, then fixed and stained for nuclei (blue), PB1 vRNA (green). Scale bar, 10 μm. Ten views were selected and vRNA numbers per cell are summarized in the graph. B: IAV disassembly was examined by staining for M1 in A549 cells that were transiently transfected with mCherry-S5. IAV WSN infection was performed with MOI = 50 for 30 min, 60 min, or 90 min. Cells were fixed and stained for nuclei (blue), M1 (white) and S5 (red). Major difference in M1 staining were pointed out with white arrow. Scale bar, 10 μm. Five views were selected and M1 staining in mCherry positive cells are summarized in the graph. (TIF) [file ppat.1010907.s006.tif]

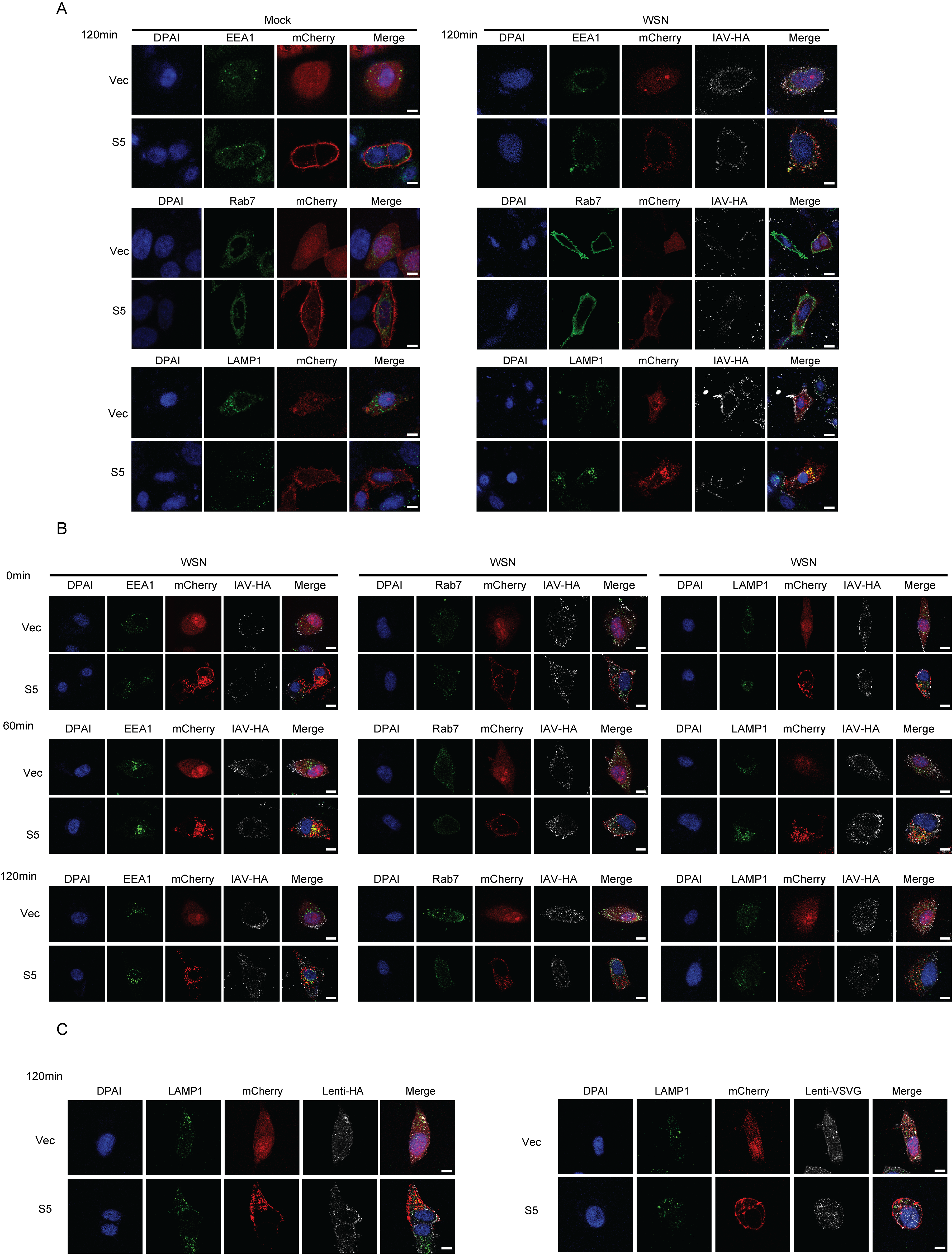

Supplement: S7 Fig — A: A549 cells were transiently transfected with mCherry-S5, then incubated with (MOI = 50) or without WSN for 2 h. Cells were fixed and stained for nuclei (blue), EEA1, Rab7, LAMP1 (green), S5 (red), HA (white). Scale bar, 10 μm. B: A549 cells were transiently transfected with mCherry-S5, then incubated with WSN at MOI = 50 for 0 h, 1h and 2h. Cells were fixed and stained for nuclei (blue), EEA1, Rab7, LAMP1 (green), S5 (red), HA (white). Scale bar, 10 μm. C: A549 cells were transiently transfected with mCherry-S5, then incubated with HA/NA or VSV-G pseudo particles (50ng p24 antigen) for 2h. Cells were fixed and stained for nuclei (blue), LAMP1 (green), S5 (red), HA/VSV-G (white). Scale bar, 10 μm. (TIF) [file ppat.1010907.s007.tif]

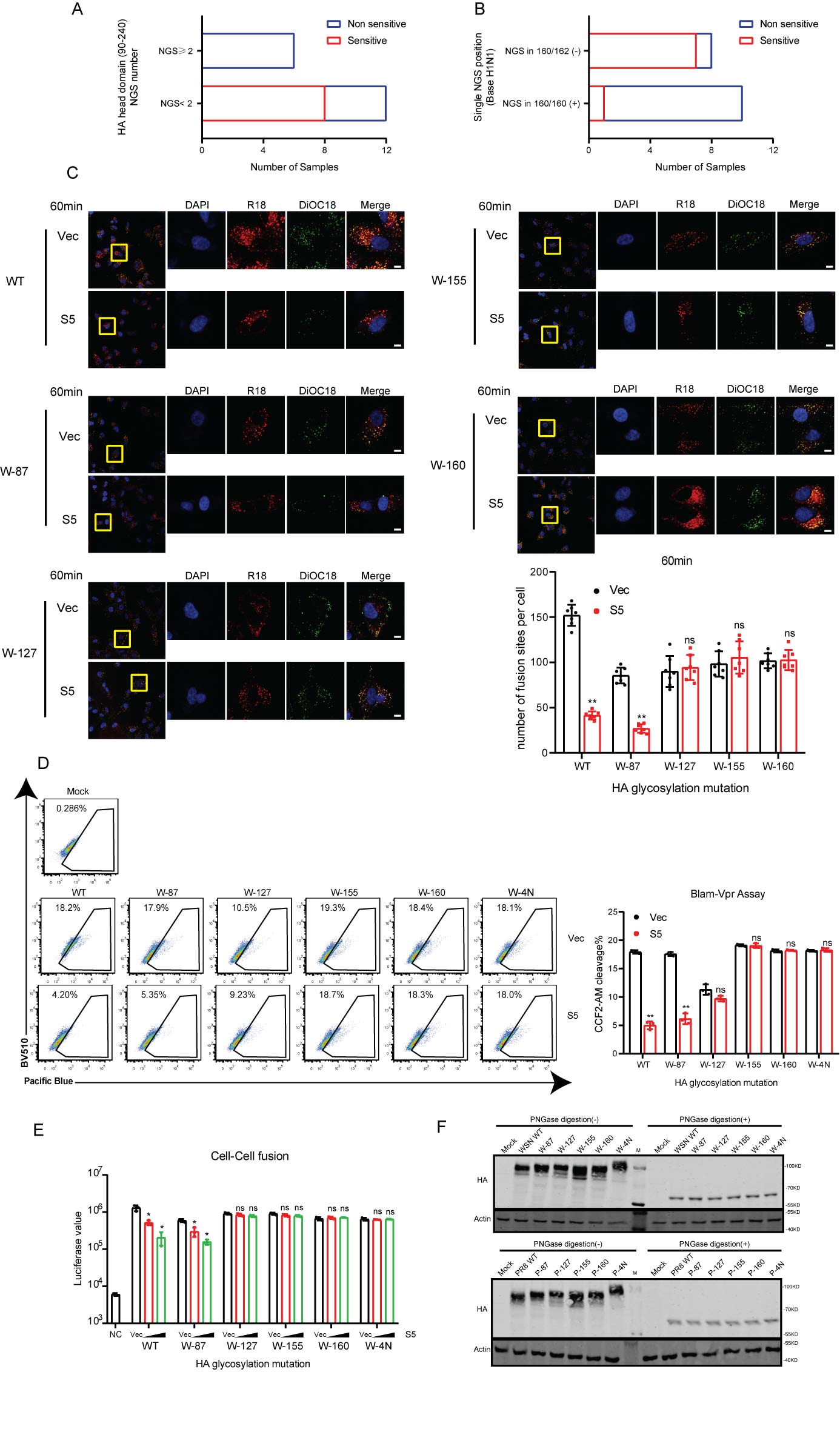

Supplement: S8 Fig — A: Statistical analysis of the N-glycosylation site numbers in HA head domain (90 to 240). B: Statistical analysis of the single N-glycosylation site in 160/162 position. C: A549 SERINC5-overexpressing cells were infected with DiOC18 and R18 labled WSN and WSN HA mutation viruses (MOI = 20), cells were fixed and stained for nuclei (blue). Virus were shown in red and virus fusion were shown in green. Scale bar, 5 μm. Seven views were selected and number of fusion sites per cell are summarized in the graph. D: BlaM assay to measure IAV fusion with target cells. A549 cells with SERINC5 overexpression (20ng p24 antigen) were incubated with IAV WSNpp that contain BlaM-Vpr for 2 h. Cells with cleaved CCF2-AM were scored with flow cytometry. Percentages of cells with CCF2-AM cleavage are summarized in the graph. E: Hela cells expressing WSN HAs and HIV-1 Tat-flag were co-cultured with 293T cells transfected with SERINC5 and LTR-Luc. Cell fusion was triggered with acidic medium. After 40 hours, cells were lysed to measure luciferase activity. NC: Negative control, 293T cells transfect with empty vector and incubated with TZM-bl cells. F: Western blot verify the glycosylation of HA mutation. 293T cells transfected with 1ug HA protein expression plasmids. 24h later cells were collected and treated with or without PNGase. Samples were resolved on 6% PAGE. Results shown are the averages of three independent experiments. Statistical significance was calculated by unpaired t-test. ns: not significant; *: P<0.05; **: P<0.01. (JPG) [file ppat.1010907.s008.jpg]

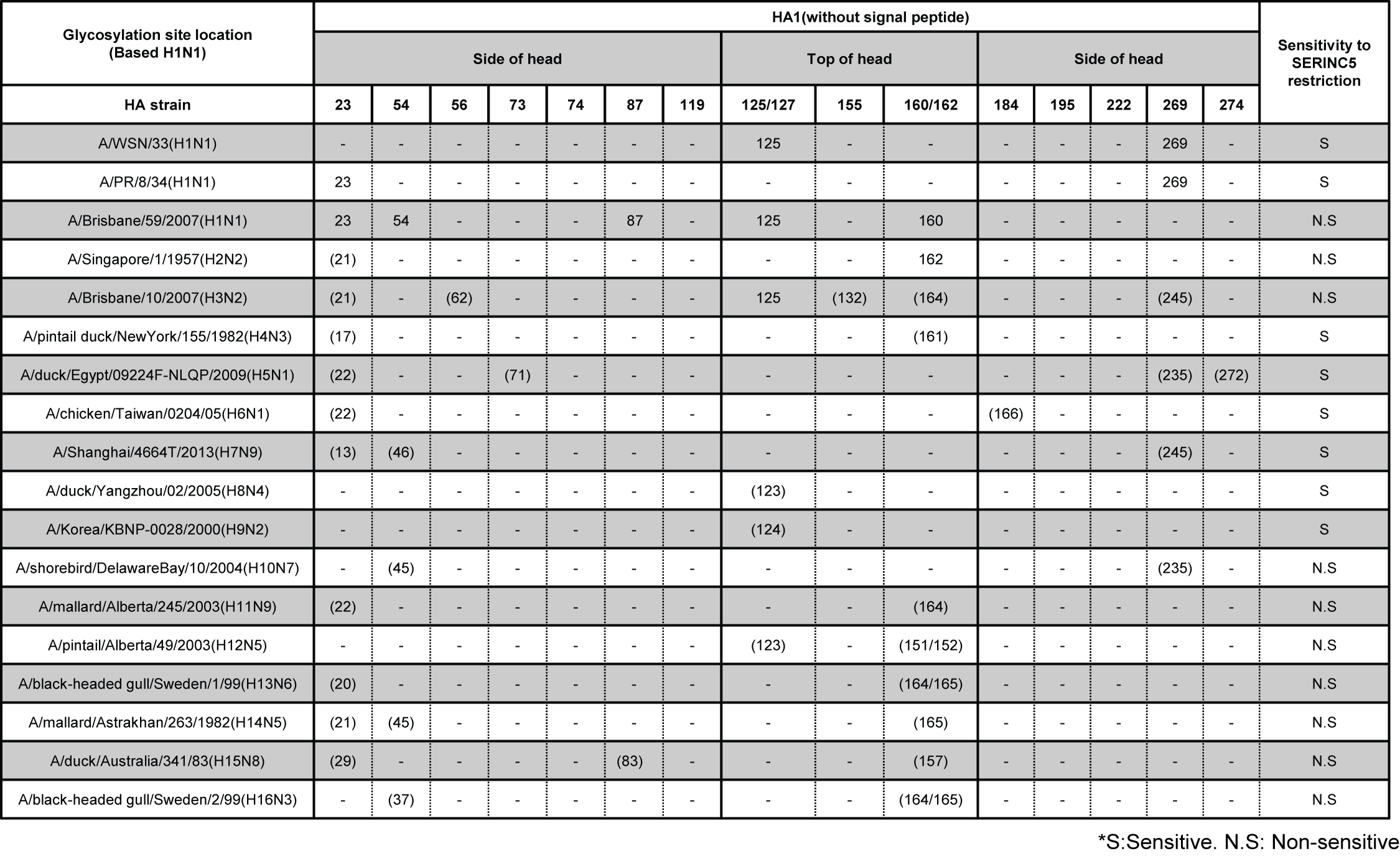

Supplement: S1 Table — (TIF) [file ppat.1010907.s009.tif]
