# Supplementary material for: SERINC5 restricts influenza virus infectivity
Source: PLoS Pathog. 2022 Oct 12;18(10):e1010907. doi: 10.1371/journal.ppat.1010907 (PMC9591065; doi:10.1371/journal.ppat.1010907)
Supplement: Attachment — Submitted filename: responses to Reviewers.doc [file ppat.1010907.s011.doc]

**Reviewer’s comments**

Dear Editor and Reviewers:

Thank you for careful reading of our manuscript and your constructive comments. We have now addressed all your comments, and revised the manuscript accordingly. The changes are included in the revised manuscript. Responses to your individual comments are listed below.

**Part I – Summary**

Please use this section to discuss strengths/weaknesses of study, novelty/significance, general execution and scholarship.

*Reviewer #1: The revised manuscript addressed most of the points suggested by reviewers. However, the authors totally misunderstood the interpretation of the PNGase F treatment. The experiment is strikingly important to support the author's argument that glycosylation regulated the sensitivity of the HA to SREINC5. Thereby, the reviewer requires a careful explanation to this issue from the authors.*

**Response:** we thank this reviewer for the insightful comments. We have now re-examined the HA mutants using a 6% PAGE (Supplement Fig 8F), and did observe clear band shift of these HA mutants. For details, please see our response in Part II.

*Reviewer #2: In my opinion, the findings of this revised manuscript are of broad interest to the field and the conclusions are well supported by the data presented.*

**Response:** we thank this reviewer for the positive comments and recognition of our study.

**Part II – Major Issues: Key Experiments Required for Acceptance**

***Reviewer #1****:*

*In Fig S8, the authors concluded that the mutant HAs are appropriately glycosylated. On the other hand, there seems no apparent band shifts in mutant HAs (except W-144) compared with WT-HAs. Especially for W-4N and P4N, addition of 4 glycosylation sites should result in significant sift of bands in the WB analysis. This should indicate the introduction of the mutation did not affect the glycosylation profile of the HA. In this situation, one of the main results that that glycosylation regulated the sensitivity of the HA to SREINC5 was not convincing. The reviewer requires a careful explanation to this issue.*

**Response:** We thank this reviewer for pointing out this inconsistency. We revisited the protocols of the Western blots examining these HA mutants, and found that the PAGE was 12.5%. This high percentage polyacrylamide gel might have obscured the migration difference of large proteins such as HA (>80 kDa) and its mutants. We have thus examined WT HA and its mutants in 6% PAGE, and presented the results of Western blots in Supplement Fig 8F. The 87, 127, 155 and 160 HA mutants, which gained one new glycosylation site, showed apparent band shift to higher molecular weights, and a much larger band shift was seen for the 4N mutants which have four new glycosylation sites. After PNGase treatment, the WT HA and all mutants were detected at the same band position in the Western blots, demonstrating that the band shift of the HA mutants was caused by glycosylation. We have made these changes in our results in lines355-362.

We also observed slight differences in the migration between the 87, 127, 155, and 160 HA mutants which all gained one new glycosylation site. This might be a result of different carbohydrate chains that might have been added to each of these new sites, in light of the reported high mannose, hybrid type and complex chains detected in HA1 (PMID: 7218432, PMID: 7305969). We have made these changes in our discussion in lines446-452. These data support the conclusion that these HA mutants gain more glycosylation and exhibit different levels of resistance to SERINC5 inhibition.

*Legends for the Fig S8F (L1046¬–1050) are inappropriate. There should not be “average of three independent experiments” nor “statistical significance”.*

**Response:** The statement “average of three independent experiments or statistical significance” was meant for the entire figure, instead of a single panel. We have corrected this error.

**Part III – Minor Issues: Editorial and Data Presentation Modifications**
***Reviewer #1:***

*Throughout the manuscript, the reviewer strongly encourages the authors to use the so-called “H3 numbering (based on Wilson et al., 1981)” system for the numbering of the HA protein,* [*https://www.nature.com/articles/289366a0*](https://www.nature.com/articles/289366a0) *.In this system, the amino acid corresponding to the matured (without signal peptide) HA of A/Hong Kong/68 (H3N2) will be used for the counting of the position.*

**Response:** We have now used the H3 numbering without signal peptide system for the numbering of the HA protein. We have made these changes in our manuscript and the figures.

*L71–72
It seems that RD114 is the abbreviation for the strain name, H7 should be subtype and RABV and LCMV should be the name of viruses. Use the abbreviations in the same category in these parentheses.*

**Response:** We have made the revision in lines 71 to 72, “Beside HIV-1, pseudo viruses with envelope proteins from feline endogenous retrovirus (RD114), influenza A virus (H7), rabies virus and lymphocytic choriomeningitis virus was shown to be restricted by SERINC5.”

*L129 (NA.) should be (NA).*

**Response:** We have made this correction.

*L207
MAL is normally used for the abbreviation for MAA-I lectin (Maackia amurensis leucoagglutinin). Maackia amurensis lectin II should be abbreviate to MAL-II, MAA-II or MAH.
https://pubmed.ncbi.nlm.nih.gov/21863598/*

**Response:** We have made this correction in line 207.

*L443–453
Using the word Env here is confusing. Do they mean Env protein of Retrovirus, or simply viral envelope protein? Please specify.*

**Response:** In lines 443 to 453, Env means viral envelope protein. We have now made this correction.

*L686–687
Text garbling in the review PDF.*

**Response:** We have made this correction.

*Fig 3D/S8C
Number of fusion site per what?*

**Response:** Number of fusion sites was determined per cell. We have made this clarification in line 886, line1042, and figure 3D/S8C.
