# Supplementary material for: SERINC5 restricts influenza virus infectivity
Source: PLoS Pathog. 2022 Oct 12;18(10):e1010907. doi: 10.1371/journal.ppat.1010907 (PMC9591065; doi:10.1371/journal.ppat.1010907)
Supplement: Attachment — Submitted filename: responses to Reviewers0706.doc [file ppat.1010907.s012.doc]

**Reviewer’s comments**

Dear Editor and Reviewers:

Thank you for careful reading of our manuscript and your constructive comments. We have now addressed all your comments, and revised the manuscript accordingly. The changes are included in the revised manuscript. Responses to your individual comments are listed below.

**Part I – Summary**

Please use this section to discuss strengths/weaknesses of study, novelty/significance, general execution and scholarship.

*Reviewer #1: The authors re-conducted the experiment to confirm the HA glycosylation; however, the result was not fully convincing. Furthermore, there is a critical concern on FigS8F that was newly provided. Thus, the reviewer again requires a careful explanation of the result from the authors.*

**Response:** we thank this reviewer for the insightful comments. For details, please see our response in Part II.

**Part II – Major Issues: Key Experiments Required for Acceptance**

***Reviewer #1****:*

*Although the authors mentioned that the experiment in Fig S8F, which was done on 12.5 % acrylamide gel, was re-conducted on 6% acrylamide gel, the pannels on "PNGase digestion(+)" seem copy-and-paste from the revision 1 figures. There is no description that the experiments on "PNGase digestion (-)" and "PNGase digestion(+)" were conducted under different conditions. Also, scales for the molecular weight in the R1 figure and R2 figure are labeled differently; in the R1 version, bands for deglycosylated HAs came under the 55KD line, but in the R2 version, they came between the 70KD and 55KD lines.*

*This might be a simple mistake by the authors; however, the authors should carefully explain this dubious result.*

*To make the situation clear, the reviewer requires the authors to provide the original (unedited) picture of the western-blotting membranes of this experiment.*

*From the amino acid sequence, the W-127 mutant lost the glycosylation site on 125 and gained an additional glycosylation site on 127. Accordingly, the total number of the glycosylation sites was unchanged. On the other hand, the clear band shift was observed even in this strain. It seemed unlikely that the difference was due to the difference in glycan structures between WT and W127 because the sift in W-127 was larger than those in W-87 and W-155. If the authors claim that site 125 was not glycosylated, the authors should demonstrate it by the site-specific glycoproteomic analysis or refer the appropriate citation.*

**Response:** We thank this reviewer for pointing out this inconsistency. We have checked the original results in the R1 version, bands for deglycosylated HAs are positioned between protein markers 55 KD and 70 KD, which was incorrectly labeled in the R1 version. We have now presented the original data of these Western blots, in which cell lysates were separated in 12.5% PAGE.

We have also presented below the original Western blots of WSN and PR8 in the R2 version. In this experiment, HA samples were separated in 6% PAGE, actin was detected in a 12.5% PAGE.

We apologize for the confusion of HA glycosylation, we have now clarified the different conditions of glycosylated and deglycosylated HAs in our manuscript in lines1083-1084.

Based on the prediction by the glycosylation sequons, WSN N142 (N125 without the signal peptide) is a potential glycosylation site. Tandem mass spectrometry analysis of A/New Caledonia/20/1999 showed a vast heterogeneity of glycan compositions at the N142 (N125 without signal peptide) position (PMID: 29048990), which supports glycosylation of WSN N142 (N125 without the signal peptide). It needs further studies to explain the band shift of 127 WSN HA mutant. We have discussed in lines451-457.

(Prediction of WSN HA glycosylation sequons)
