# Supplementary material for: SERINC5 restricts influenza virus infectivity
Source: PLoS Pathog. 2022 Oct 12;18(10):e1010907. doi: 10.1371/journal.ppat.1010907 (PMC9591065; doi:10.1371/journal.ppat.1010907)
Supplement: Attachment — Submitted filename: responses to Reviewers.doc [file ppat.1010907.s013.doc]

**Reviewer’s comments**

Dear Editor and Reviewers:

Thank you for careful reading of our manuscript and your constructive comments. We have now addressed all your comments, and revised the manuscript accordingly. The changes are included in the revised manuscript. Responses to your individual comments are listed below.

**Part I – Summary**

Please use this section to discuss strengths/weaknesses of study, novelty/significance, general execution and scholarship.

*Reviewer #1: Although the authors revised the descriptions on the SFig. 8 (PNGase digestion), the settings for this experiment were not appropriate.*

**Response:** we thank this reviewer for the insightful comments. For details, please see our response in Part II.

**Part II – Major Issues: Key Experiments Required for Acceptance**

***Reviewer #1****:*

*The authors resolved PNGase (+) samples with 12.5% PAGE gels, whereas PNGase (-) samples with 6% gels.*

*Resolving the protein with 12.5% gels underestimates the electrophoresis mobility of proteins; this should be why the authors used 6% gels for resolving PNGase (-) samples.*

*Accordingly, the reviewer again requires the authors to conduct the western blot analysis of both PNGase (+) and (-) samples on 6% PAGE gels. Also, the pairs of PNGase (+) and (-) samples should be resolved strictly on the same gels.*

**Response:** We thank this reviewer for pointing out this disadvantage. We have resolved the PNGase(+) and PNGase(-) HA mutants samples on the same 6% PAGE gels. The original data of these Western blots were presented here.

We also clarified the conditions of glycosylated and deglycosylated HAs in our manuscript in line1083.
