# Supplementary material for: SERINC5 restricts influenza virus infectivity
Source: PLoS Pathog. 2022 Oct 12;18(10):e1010907. doi: 10.1371/journal.ppat.1010907 (PMC9591065; doi:10.1371/journal.ppat.1010907)
Supplement: Attachment — Submitted filename: Reviewer comments.doc [file ppat.1010907.s010.doc]

**Reviewer’s comments**

Dear Editor and Reviewers:

Thank you for careful reading of our manuscript and your constructive comments. We have now addressed all your comments, and revised the manuscript accordingly. The changes are included in the revised manuscript. Responses to your individual comments are listed below.

**Part I – Summary**

Please use this section to discuss strengths/weaknesses of study, novelty/significance, general execution and scholarship.

***Reviewer #1:*** *The manuscripts described the roles of SERINC5, a host protein that was previously demonstrated to have anti-retrovirus activity, in influenza A virus (IAV) infection. It has been previously reported that SERINC5 inhibited virus entry of a virus harboring HA from H7 IAV (Diehl et al., 2021). Accordingly, the main findings of the study that authors argued seemed to be the following three:
- SERINC5 inhibited actual infection of AIV.
- SERINC5 inhibited virus membrane fusion steps.
- The glycosylation of HA proteins regulated the inhibition.
On the other hand, although the pseudotyped HIV-based experiments support the second argument well, the major concerns were still lying on the experiments using authentic AIVs. In addition, the mechanisms of how the glycosylation of the HA modulates SERIN5C-mediated AIV inhibition were not demonstrated nor even discussed. Accordingly, the scientific significance of the study was unclear.*

**Response:** we thank this reviewer for the insightful comments as well as the recognition of the novel findings we herein reported. We have now used the dual wavelength imaging (PMID: 16439557) to measure the effect of SERINC5 on the fusion of authentic IAV with cell membranes (Fig. 3D), the data convincingly support SERINC5 inhibits IAV infection at the membrane fusion step. We have also discussed the possible mechanisms behind the role of glycosylation in modulating HA sensitivity to SERINC5 (lines 443-453). One new finding of our study is that in addition to SERINC5 achieving inhibition of virus entry as part of virus particles, SERINC5 can also act in the virus target cells to deter the entry of incoming IAV particles (Fig. 4).

***Reviewer #2:*** *In their manuscript, Fei Zhao et al. show that SERINC5 can inhibit entry of influenza viruses. Interestingly, this inhibition can take place when SERINC5 is expressed in target or producer cells. The authors also find that different types of influenza viruses differ in their sensitivity to SERINC5 and that low glycosylation levels correlate with sensitivity. Overall, their findings are supported well by the data presented and the findings are of relevance. However, several points need to be addressed.*

**Response:** we thank this reviewer for the constructive appraisal of our study. The raised points are addressed below.

***Reviewer #3:*** *Zhao and colleagues investigated the impact of SERINC5 on influenza A virus (IAV) infection. In brief, they report that expression of SERINC5 in (retroviral) particle producer cells and in target cells reduced particle infectivity and viral entry, respectively. Further, SERINC5 is shown to inhibit authentic IAV infection and evidence is reported that an early step, membrane fusion, is blocked. Finally, it is demonstrated that hemagglutinins (HA) from different IAV subtypes exhibit differential SERINC5 sensitivity and that the presence of certain sequons in HA can confer SERINC5 resistance. The study reports, for the first time, inhibition of IAV infection by SERINC5. It contains a good amount of largely well controlled data and is of interest to the field. On the downside, it provides little mechanistic insights. The following points should be addressed.*

**Response:** We thank this reviewer for the constructive comments. The raised points are addressed as below.

**Part II – Major Issues: Key Experiments Required for Acceptance**

***Reviewer #1****:*

*BlaM-Vpr pseudovirus fusion assay demonstrated that virus-mediated membrane fusion was inhibited in pseudotyped HIV. On the other hand, the redistribution of M1 was not fully convincing to support the Authors’ arguments. The approach is not a widely accepted method for visualizing the AIV fusion process. The authors should validate the method with appropriate control studies or provide appropriate references for the method. Alternatively, the authors should use more widely accepted methods to evaluate the AIV fusion process.*

**Response:** we agree that M1 redistribution is not widely used to measure IAV entry and uncoating. This method was reported in a study aimed at examining the effect of a host factor EPS8 on IAV entry and uncoating (PMID: 31747592). We did observe that SERINC5 reduced M1 redistribution in IAV entry experiment by quantifying M1 signal in cells from five views. This result is now presented as a supplemental figure (S6 B). We revised the text accordingly in line 211

In the meantime, we used the dual wavelength imaging method to measure the effect of SERINC5 on IAV entry and membrane fusion as described in PMID: 16439557 and PMID:25031340. Two lipophilic probes, DiOC18 (Green) and R18 (Red), were used to label the membrane of IAV particles. The results showed that the SERINC5-expressing cells was much less fused with IAV particles compared with the control cells not expressing SERINC5. This new data is presented in Fig 3D, described in lines 234-243.

*The authors argued that the number of the N-linked glycosylation sites is related to the susceptibility toward SERINC5-mediated inhibition. However, this was not supported by the following experiment. In WSN-144, the original N-glycosylation sequon at N142 was abrogated, and alternatively, the virus acquired N-glycosylation sequon at N144. Accordingly, the total numbers of the putative N-glycosylation were the same in WT-WSN and WSN-144. Nevertheless, WSN-144 was resistant to SERINC5 inhibition. How do the authors discuss this discrepancy?*

**Response:** we do observe a correlation between the number of glycosylation sites and sensitivity of HA to SERINC5 inhibition by analyzing 18 types of HA (Fig. 4 S1 Table). This reviewer rightfully pointed out that some HA subtype may not necessarily comply with this correlation such as WSN-144 vs WSN-WT. One explanation is that in addition to the number of glycosylation sites, the position may also matter in terms of determining the sensitivity to SERINC5. For example, N144 is located to the center top of HA head whereas N142 is located at the edge. It is possible that higher degree of glycosylation at the center top of HA head confers more resistance to SERINC5 restriction. This possibility is now discussed in lines336-339.

*Although the authors argued that the glycosylation of HA proteins regulated the SERINC5-mediated inhibition, the underlying mechanisms were totally unknown. Membrane fusions are mediated by HA2, not HA1, subunit of the HA. How does the glycosylation of the globular head domain of the HA regulate the interaction with host factors that interfere membrane fusion process? The authors should investigate whether the membrane fusion process was interfered in glycosylation mutants and discuss the potential mechanisms underlying.*

**Response:** we have now measured the membrane fusion activity of these HA glycosylation mutants and their responses to SERINC5 restriction, using the dual wavelength imaging method, the BlaM assay, and the cell-cell fusion system. Results are shown in S8C-E, and show that except for WSN-144, all the other glycosylation mutants resist SERINC5 inhibition. This data is described in lines 350-356. The potential mechanisms are now discussed in lines 443-453.

*The reviewer wondered about some discrepancies in the control studies. For example, in Fig 1D, virus growth reached 10^7 pfu/mL in control cells (empty vector-transfected cells). On the other hand, in Fig 1E, that reached only 10^5 in control KO cells, although the experimental conditions of both experiments did not differ so much. Similar tendencies were also observed in Fig2 H and I, and Blam-Vpr Assay in Fig 3E and F. Authors should explain why these discrepancies occurred?*

**Response:** we thank this reviewer for pointing out these discrepancies. We have now indicated in the legends of these figures the MOIs of IAV that were used for the infections. Different MOIs have led to the different viral yields which are reflected on the scales of the Y axis.

*In some figures, the scaling is inappropriate. For example, the scaling of Fig 1A ranged in 10^4 to 10^9. Those ranged in 10^4 to 10^6 in Fig 2H, whereas in 10^5 to 10^7 in Fig 2I. These scales inappropriately exaggerate the difference between the two groups, thereby would mislead the readers.*

**Response:** We have now corrected these scales.

*The authors evaluate the expression of sialosides using SNA staining to ensure that the expression of SERINC5 did not affect the receptor expression. Laboratory strains of IAV such as WSN or PR8 often showed altered receptor binding specificity from original human isolates. It should be doubtful whether WSN utilized only 2-6 sialosides for the infection. The authors should evaluate receptor binding preference of WSN, which was used in the present study, or alternatively evaluate the distribution of 2-3 sialosides.*

**Response:** we have now examined the distribution of 2-3 sialosides using biotinylated *Maackia amurensis* lectin II (MAL, Vectore Labs #B-1265) as described in PMID: 22046135, followed by detection with streptavidin-FITC. The results are presented in S5A, and show that SERINC5 expression has no effect on the distribution of 2-3 sialosides. Please see line 207.

*The authors should check whether each of the glycosylation variants properly acquired glycosylation, not potential glycosylation sites, by using PNGase-digestion and western blotting.*

**Response:** we have now used PNGase and Western blotting to examine glycosylation of HA mutants. The results showed that WSN and PR8 HA were both properly glycosylated (S8F).

**Reviewer #2**:

*- Fig. 1: SERINC5 seems to be incorporated in all types of VLPS except the IAV-VLPs. However, in these samples also cellular levels of SERINC5 are lower. This should be examined more closely. Does the expression of HA/NA lead to reduced SERINC5 levels or is this a transfection issue in this particular experiment?*

**Response:** we repeated the experiments in Fig 1C and did not observe downregulation of SERINC5 expression by HA/NA. We further confirmed our results by co-transfection SERINC5 with HA/NA. Western blot is shown below.

*- Supp. Fig. 5: The assays shown here need a control to show that the assays would pick up a decrease. This is particularly important for quantifying virus binding by immunofluorescence staining of HA. If the input of virus is halved would that be detected in this assay?*

**Response:** we have repeated the virus binding experiment with a lower MOI=5, and did not observe any effect by SERINC5. The data are shown in S5B.

*- Fig. 3 C-D only show very few cells as examples. These data should be supported by a quantitative analysis on many cells.*

**Response:** we have quantified vRNA in cells from 10 views and presented the results in Fig 3C and S6.

*- Fig. 4A: Only relative luciferase levels are shown. Did inhibition correlate with infectivity of the VLPs? Absolute luciferase values should be included as supp. fig..*

**Response:** we have now presented the original luciferase values in S5E-F, to further support our conclusions.

*- The authors show convincingly that the level of HA glycosylation determines SERINC5 sensitivity but this exciting finding is not tested for HIV. Given that the authors already have established the HIV VLP system they should test different variants of env that differ in glycosylation. It will strengthen the manuscript if the authors can show that the level of glycosylation matters also in inhibiting other viruses.*

**Response:** we thank this reviewer for this valuable suggestion. We felt that the primary focus of this manuscript is to demonstrate SERINC5 restriction of IAV at the membrane fusion step, including HIV data is beyond this scope and very likely dilutes this focus. We are working on this important subject and hope to report the findings in a separate manuscript.

*- The discussion section is mostly a repetition of the results but no hypothesis on the mechanism is provided. This needs to be revised and a testable hypothesis for the mechanism of action should be included.*

**Response:** we have now revised our discussion in lines 399-416, 443-464 and put forward a few possible mechanisms accounting for the role of glycosylation in modulating HA sensitivity to SERINC5.

***Reviewer #3****:*

*Figure 3G (cell-cell fusion) is central to the manuscript and should be better controlled. Please include control viral envelope proteins (for instance HIV-Env and VSV-G), in order to demonstrate specificity, and please include a negative control (empty vector), which allows the reader to judge the assay background.*

**Response:** we have now repeated the cell-cell fusion experiments by including the control viral glycoproteins of HIV-1 envelope and VSV-G, as well as the empty vector as the negative control (Fig 3G).

*Supplemental figure 6B is unexpected and the data should be bolstered by additional analyses. Thus, please examine the kinetics of this effect and please determine whether this effect is also seen with retroviral particles bearing HA but not VSV-G.*

**Response:** we have now examined the kinetics of SERINC5 translocation to endosomes and lysosomes. The results are presented in S7. We found that WSN infection caused re-localization of SERINC5 from the plasma membrane to endosomes and lysosomes. HA-pseudotyped, but not VSV G-pseudotyped, retroviral particles can cause SERINC5 re-localization to endosomes and lysosomes.

*Little information is available on the expression of SERINC5 in relevant IAV target cells. Is SERINC5 expressed in the respiratory epithelium? Is expression upregulated by IFN and if so, does SERINC5 knock-down render IAV infection less susceptible to inhibition by IFN?*

**Response:** Endogenous SERINC5 has not been detected in the respiratory epithelial cells with commercial antibodies. SERINC5 expression was not inducible by interferon nor LPS (PMID: 26416734). However, SERINC5 was reported to enhance MDA5-mediated IFN signaling (PMID: 33013817), and can interact with MAVS and TRAF6 to inhibit HIV-1 infection (PMID: 34520227).

*It should be discussed whether also for HIV Env the number of sequons correlates with SERINC5 sensitivity. Furthermore, it should be discussed whether IFITM3 sensitivity of HAs (see for instance PMID: 21253575) from different IAV subtypes correlates with SERINC5 sensitivity.*

**Response:** HIV-1 Env is heavily glycosylated (PMID:2355006). Glycosylation can protect Env from binding by neutralizing antibodies, modulate Env conformations, and can even become an epitope of host antibodies. Env of different HIV-1 strains are quite divergent, often vary in the number and position of glycosylation sites likely as a result of evading host immunity from both the adaptive and innate arms including host restriction factors like SERINC5. It will be informative to compare the glycosylation profiles of different HIV-1 Env glycoproteins and their sensitivities to SERINC5 inhibition. The findings are expected further strengthen the conclusion of this study on modulation of SEINC5 restriction by Env glycosylation. We have discussed in lines 456-464.

Both IFITM3 and SERINC5 impair virus entry, it will be highly interesting to examine and correlate the sensitivities of different HA subtypes to these two antiviral factors. In the literature, some HA types, such as HA1, HA3, HA5 and HA7 were tested against IFITM3, and were all inhibited. No study has comprehensively tested the full spectrum of HA types against IFITM3 as we have done in this study against SERINC5. We are in the process of examining IFITM3 inhibition of the HA clones we have in hand, and hopefully will be able to identify any correlation with SEIRNC5 and any possible role of glycosylation in response to IFITM3 as we observed here with SERINC5. We have discussed in lines 399-416.

**Part III – Minor Issues: Editorial and Data Presentation Modifications**
***Reviewer #1:***

*L244 based on the antigenicity of the HA and NA.*

**Response:** We have made this correction.

*L243 not seasonal H1N1 IAV, but Russian-flu H1N1 strain.*

**Response:** We have corrected this error.

*L324 MLV and EIAV, spell out.*

**Response:** We have provided the full names, murine leukemia virus (MLV), equine infectious anemia virus (EIAV).

***Reviewer #2:***

*- L. 114 „IAV membrane also carries the M1 and M2 proteins” This statement suggests that M1 is embedded in the IAV envelope but it functions as a matrix protein and is not “carried” by the IAV membrane. This should be reworded.*

**Response:** We have revised this sentence as, “The viral membrane is embedded with the M2 ion channel protein, and the hemagglutinin (HA) and neuraminidase (NA.) Inside the virion, viral ribonucleoprotein (vRNP) complexes associate with the viral matrix protein M1 which associates with the inner leaflet of viral membrane. M1 participates in viral particle assembly and can bind to the cell plasma membrane and/or the cytoplasmic tail of viral transmembrane proteins”.

*- L. 178-182 A reference should be given for WSN-Gluc.*

**Response:** We have now provided the reference in line 179.

*- Fig. 2 H-I: In my opinion, “defective virus” is not a suitable heading for these panels. I would say WSN-Gluc.*

**Response:** We have now used WSN-Gluc as suggested.

*- L. 275 In my opinion it is confusing to introduce the abbreviation NGS for N-glycosylation site as NGS usually means next generation sequencing. I would spell out N-glycosylation site.*

**Response:** We have now only used N-glycosylation site in the manuscript.

***Reviewer #3:***

*Why are SERINC5 levels low upon coexpression of WSN HA/NA (figure 1C)*

**Response:** we have now repeated this experiment and confirmed no significant change in SERINC5 level with WSN HA/NA expression (Fig 1C). We further confirmed our results by co-transfection SERINC5 with HA/NA. Western blot is shown below.

*“IAV membrane also carries the M1 and M2 proteins.” M1 is not an integral membrane protein.*

**Response:** We have now revised this sentence as, “The viral membrane is embedded with the M2 ion channel protein, and the hemagglutinin (HA) and neuraminidase (NA.) Inside the virion, viral ribonucleoprotein (vRNP) complexes associate with the viral matrix protein M1 which associates with the inner leaflet of viral membrane. M1 participates in viral particle assembly and can bind to the cell plasma membrane and/or the cytoplasmic tail of viral transmembrane proteins”.

*Ideally, figures 5B-D should include controls like neuraminidase treatment. At least, the absence of such controls should be stated.*

**Response:** HA mutants were used to pseudotype lentivirus particles that were produced with WSN-NA. We have made this correction in line 917.
